# DHX36 modulates stress granule assembly independent of recruitment of mRNAs with G-quadruplex sequence motifs

**DOI:** 10.1093/nar/gkaf938

**Published:** 2025-09-23

**Authors:** Li Yi Cheng, Nina Ripin, Thomas R Cech, Roy Parker

**Affiliations:** Department of Biochemistry, University of Colorado Boulder, Boulder, CO 80303, United States; Department of Biochemistry, University of Colorado Boulder, Boulder, CO 80303, United States; Department of Biochemistry, University of Colorado Boulder, Boulder, CO 80303, United States; Howard Hughes Medical Institute, University of Colorado Boulder, Boulder, CO 80303, United States; Department of Biochemistry, University of Colorado Boulder, Boulder, CO 80303, United States; Howard Hughes Medical Institute, University of Colorado Boulder, Boulder, CO 80303, United States

## Abstract

Stress granules are RNA–protein condensates that form in response to an increase in untranslating mRNPs (messenger ribonucleoproteins). Stress granules form by the condensation of mRNPs through a combination of protein–protein, protein–RNA, and RNA–RNA interactions. Several reports have suggested that G-rich RNA sequences capable of forming G-quadruplexes (rG4s) promote stress granule formation. Here, we provide three observations arguing that G-tracts do not promote messenger RNA (mRNA) accumulation in stress granules in human osteosarcoma cells. First, we observed no difference in the accumulation in stress granules of reporter mRNAs with and without G-tracts in their 3′ UTRs. Second, in U-2 OS cell lines with reduced expression of DHX36, which is thought to unwind G-quadruplexes, the accumulation of endogenous mRNAs was independent of their predicted rG4-forming potential. Third, while mRNAs in stress granules initially appeared to have more rG4 motifs than bulk mRNAs, this effect disappeared when rG4 motif abundance was normalized to mRNA length. However, we observed that in a G3BP1/2 double knockout cell line, which strongly inhibits stress granule formation, reducing DHX36 expression rescued stress granule-like foci formation. This indicates that DHX36 can limit stress granule formation, potentially by unwinding *trans*-rG4s or limiting other intermolecular RNA–RNA interactions that promote stress granule formation.

## Graphical Abstract

**Graphical Abstract:**
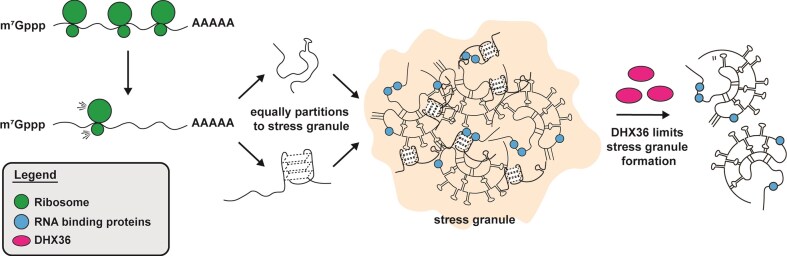

## Introduction

Stress granules are membrane-less cytoplasmic messenger ribonucleoprotein (mRNP) assemblies that form upon various types of cellular perturbation, such as oxidative stress and heat shock. Stress granules are thought to form from the summation of protein–protein, protein–RNA, and promiscuous RNA–RNA interactions when messenger RNA (mRNA) translation is inhibited and ribosomes run off mRNAs [1–4]. We have previously demonstrated that stress granules contain molecules from essentially all mRNA species found in the cytoplasm, with no single mRNA or non-coding RNA making up >1% of the stress granule RNA content [5]. Stress granule formation is promoted by an excess of free untranslating mRNAs [6] and is inhibited by drug treatments that trap ribosomes on mRNAs [7]. These observations suggest that concentration-driven condensation of ribosome-free and translationally inactive mRNAs forms the basis for stress granule assembly.

RNA serves two roles in the formation of stress granules. First, as a critical component of stress granules, RNA can serve as a scaffold to recruit RNA-binding proteins that promote stress granule formation. For example, deletion of the G3BP1 RNA binding domain abolishes stress granule formation in cells [8, 9], suggesting that G3BP1 is required to bind RNA to promote stress granule formation [8–10]. RNA binding can also recruit “client proteins,” which are RNA-binding proteins that localize to stress granules through binding resident RNAs [11].

In a second role, RNA can facilitate stress granule formation through enhancing RNA condensation. *In vitro*, we previously demonstrated that RNA self-assemblies made from total yeast RNA have a transcriptome similar to that of endogenous stress granules [12]. Similar work by others has also shown that such RNA self-assemblies can be formed more readily when the trigger RNA species can engage in stable intermolecular interactions [13, 14]. Direct evidence for intermolecular RNA–RNA interactions promoting RNP granules comes from the observations that specific base-pairing in *trans* between *oskar* mRNAs or between *bicoid* mRNAs is required for their recruitment to RNP granules during *Drosophila* oocyte development [15–17]. During cellular stress, mRNA sequences that are normally protected by ribosomes become exposed, increasing their propensity to form promiscuous intermolecular RNA–RNA interactions to enhance mRNP granule formation [18].

One type of RNA structure that has been proposed to contribute to stress granule formation is the RNA G-quadruplex (rG4). rG4s are composed of two or more stacked guanine quartets, each formed by the planar interaction of guanine bases via Hoogsteen base pairing. *In vitro*, rG4 sequences can undergo liquid–liquid phase separation and form droplets [19, 20]. Such rG4 assemblies could help to bring together a high local concentration of RNAs and/or rG4 binding proteins to favor assembly formation in cells.

Several studies have proposed that rG4s promote stress granule formation. This was first suggested by the observation that oligonucleotides that can form rG4s can promote RNP condensation *in vitro* and stress granule formation when transiently transfected into cells [13]. In addition, several high-throughput transcriptomic studies have revealed numerous putative rG4-forming sequences in mRNAs [21–23]. Moreover, mRNAs with putative rG4 sequences were suggested to accumulate in stress granules to a greater extent than mRNAs without predicted rG4 sequences [23]. More recently, intermolecular rG4s have been proposed to bind proteins to nucleate stress granule formation. Specifically, interaction of rG4s with RNA-binding proteins such as DNAPTP6 [24] and SERF2 [25] was suggested to coordinate stress granule assembly in mammalian cells.

The possible role of rG4s in stress granules raises the possibility that the DEAH-box helicase DHX36, which is the predominant protein known to resolve rG4s in HeLa cells [26], regulates stress granule formation by targeting and unwinding endogenous rG4s [27]. *In vitro* biochemical studies showed that DHX36 exhibits tight binding affinity with rG4s in the sub-nanomolar range [28] and preferentially unwinds rG4s relative to other nucleic acid secondary structures [26, 29]. In cells, loss of DHX36 was shown to increase the abundance of G-rich, translationally inactive DHX36 target mRNAs capable of forming rG4s [23]. Moreover, DHX36 is found within stress granules upon cellular stresses [23, 30, 31]. Precedent for how DHX36 might affect stress granules comes from studies on eIF4A, which can limit stress granule formation by limiting intermolecular RNA–RNA interactions [32]. DHX36 might affect stress granule formation by preventing rG4s from forming in mRNAs that serve as binding sites for rG4-binding proteins that promote stress granules. Alternatively, DHX36 could limit the formation of rG4s in *trans* between multiple RNAs, which then could contribute to intermolecular RNA–RNA interactions that stabilize stress granule assembly.

Herein, we examined whether guanine-tract (G-tract) sequences with quadruplex-forming potential have any role in targeting mRNAs into stress granules in human U-2 OS cells. We report several key observations. First, mRNA accumulation to stress granules is independent of G-tracts in several tested mRNAs. Second, the accumulation of endogenous mRNAs with putative rG4 motifs into stress granules is unchanged upon DHX36 knockdown, and their localization to stress granules is more length-dependent than rG4-dependent. Third, we observe that DHX36 can limit stress granule formation, perhaps by resolving *trans* rG4s or other types of intermolecular RNA–RNA interactions.

## Materials and methods

### Circular dichroism

RNA was purchased from Integrated DNA Technologies (IDT) and dissolved in double-distilled water. For CD, RNA was adjusted to 10 mM Tris (pH 7.5) with or without 100 mM KCl or LiCl as indicated. The 150 μL sample (∼0.5 mg/mL RNA) was heated to 95°C for 5 min and snap cooled. Either KCl buffer or LiCl buffer alone was used as a baseline control for each salt condition. CD spectra were collected with Chirascan Plus Circular Dichroism and Fluorescence Spectrometer (Applied Photophysics) using a 0.5 mm path length cell. Spectra were recorded from 360 to 200 nm with a 0.5 nm step size and 0.5 s integration time. When switching between samples, the cuvette was washed 2× with water and once with 100% ethanol. Baselines were subtracted from spectra and CD is reported in mean residue molar ellipticity.

### Cell culture

Human osteosarcoma U-2 OS cells were maintained in Dulbecco’s modified Eagle’s medium (DMEM) supplemented with 10% fetal bovine serum and 1% penicillin/streptomycin at 37°C/5% CO_2_.

### Cell lines and plasmids

Wild-type (WT) and G3BP1/2 double knockout (dKO) U-2 OS were kindly provided by Paul Anderson’s Lab at Brigham and Women’s Hospital, Boston, MA, USA [8]. Tet-inducible luciferase reporter was a gift from Moritoshi Sato (Addgene plasmid #64127; http://n2t.net/addgene:64127; RRID: Addgene_64127, [33]).

Various G-tract sequences were synthesized by GenScript (Supplementary Table S1 for sequences) and cloned into the 3′ UTR of the luciferase reporter via InFusion cloning. 1× reporter constructs were dual luciferase reporter constructs with firefly luciferase (FLuc) as an internal control. Both renilla luciferase (RLuc) and FLuc were controlled by the Tet-inducible promoter, independently. 5× reporter constructs only had RLuc upstream of rG4 motifs; it did not contain FLuc as an internal control.

To integrate the luciferase reporter constructs into the *AAVS1* safe harbor locus, WT U-2 OS cells were transfected with 1 μg CRISPR/Cas9 plasmid (pRP2854) in conjunction with 1 μg appropriate luciferase reporter construct using JetPRIME transfection reagent (VWR Scientific, 89129-922). Transfection of pRP2854 alone was used as a negative control. Twenty-four hours following transfection, cells were split from a six-well plate to a T25 flask with media containing 1 μg/mL puromycin (Sigma–Aldrich, P9620) to begin selection for cells with genomic integration. Following 24 h with puromycin selection, media was replaced with fresh puromycin. After all cells were dead in the negative control plate, media was replaced with fresh media lacking puromycin for 48 h. Puromycin was then added for another 48 h to finalize the selection. Single colony selection was not done for these experiments.

DHX36 hypomorph (DHX36hm) constructs in WT and G3BP1/2 dKO U-2 OS cell lines were generated using methods from [34]. Briefly, two CRISPR/Cas9 guide RNAs targeting different regions within the DHX36 locus were designed using the IDT CRISPR guide target design tool. Overlapping oligos (DHX36 sgRNA 1, 2 sense and DHX36 sgRNA 1, 2 antisense [Supplementary Table S1]) were annealed in T4 DNA ligase buffer (NEB, B0202S) and ligated into the BbsI-HF (NEB, R3539S) sites in pSpCas9(BB)-2A-GFP (PX458) (48138; Addgene) using T4 DNA ligase (NEB, B0202S). To generate DHX36hm in U-2 OS and G3BP1/2 dKO U-2 OS lines, cells (T-25 flask; 60% confluent) were co-transfected with 3 μg of pSpCas9(BB)-2A-GFP-DHX36 sgRNA1 + 2 and 400 ng of pcDNA3.1-puro using 15 μl of Lipofectamine 2000 (Thermo Fisher Scientific, 11668019) according to the manufacturer’s instructions. Twenty-four hours following transfection, cells were puromycin selected as described earlier. Selective medium was replaced with normal growth medium after all cells were dead in the negative control. When cells became 80% confluent, cells were serially diluted and plated on 15 cm dishes. After visible colony formation, individual colonies were isolated, propagated, and tested for DHX36 loss via immunoblotting.

Generation of the pLenti-EF1-Blast-HA-FLAG-DHX36 lentiviral plasmids was performed as described in [35]. Briefly, untagged DHX36 sequences were amplified from Addgene plasmids #159585 and #159587, and the sequences were inserted into the XhoI/XbaI sites of pLenti–EF1-Blast vector using In-Fusion seamless cloning (Takara Bio). HEK293T cells (T-25 flask; 80% confluent) were co-transfected with 2.7 μg of pLenti-EF1-Blast-HA-FLAG-DHX36, 870 ng of pVSV-G, 725 ng of pRSV-Rev, and 1.4 μg of pMDLg-pRRE [36], using 20 μl of Lipofectamine 2000. The medium was collected 48 h after transfection and filter-sterilized with a 0.45 μm filter. Then, U-2 OS G3BP1/2dKO + DHX36hm cells (T-25 flask; 80% confluent) were transduced with 1 ml of lentiviral stocks containing 10 μg/ml of polybrene (Millipore Sigma, TR-1003-G) for 1 h. DMEM was then added to the flask after an hour. 24 h after transduction, cells were reseeded into a T-75 flask containing 10 μg/ml blasticidin (Thermo Fisher Scientific, A11139-03) selective medium. Cells were maintained in selective medium for 4–5 days before returning to normal DMEM.

### Immunoblotting

Cells were washed with ice-cold phosphate buffered saline (PBS) and lysed with Pierce RIPA Buffer (Thermo Fisher Scientific, 89900), 1* Phosstop™-phosphatase inhibitor (Roche, 4906837001), and 1* cOmplete Mini EDTA-free Protease Inhibitor Cocktail (Sigma–Aldrich, 11836170001). Cells were lysed on ice for 15 min (flicked every 5 min) and then clarified by centrifugation at 4°C, max speed for 10 min, in a benchtop centrifuge (Benchmark Scientific). 4× Nu-PAGE LDS sample buffer (Thermo Fisher Scientific, NP0007) was added to lysates to a final concentration of 1×; samples were heated for 10 min at 70°C and then loaded into 4%–12% Bis-Tris Nu-PAGE gel (Thermo Fisher Scientific, NP0336BOX) and transferred with IBlot™ 2 PVDF transfer stacks (Thermo Fisher Scientific, IB24002). Membranes were blocked with 5% nonfat dry milk (Bio-rad, 1706404) in Tris-buffered saline with 0.1% Tween-20 (Sigma–Aldrich, P9416) (TBST) for 1 h and then incubated with primary antibody in TBST for 1 h at room temperature or overnight at 4°C. Antibody dilutions are listed in Supplementary Table S1. Membranes were washed 3× with TBST and then incubated with secondary antibody at room temperature for 1 h in TBST. Membranes were washed 3× again in TBST, and antibody detection was achieved with chemiluminescence substrate (Thermo Fisher Scientific, 34095).

### Stress conditions

To induce stress granules, cells were treated with 500 μM sodium arsenite (Sigma–Aldrich S7400) in DMEM for 1 h at 37°C unless otherwise specified. Cells were fixed after the completion of stress with 4% paraformaldehyde. To examine stress recovery, cells were incubated with 300 μM sodium arsenite in DMEM for 1 h before washing with PBS and replacing with normal DMEM.

### Dual luciferase assay

U-2 OS cells were plated at 50% confluency in 96-well plates for luciferase assay. On the next day, cells were lysed using the Passive Lysis Buffer (Promega). RLuc and FLuc activities were measured using the Dual-luciferase Reporter Assay (Promega) as per manufacturer’s protocol with the CLARIOstar^®^ Plus Multi-mode Microplate Reader. WT U-2 OS cells with no reporter constructs were used as the negative control for background luciferase activity. In all cases, RLuc values were normalized to FLuc values.

### siRNA-mediated knockdown

For siRNA knockdowns, 200 000 cells per well were seeded into six-well plates. After 24 h, cells were transfected with 20 nM siGENOME SMARTpool siDHX36 (Dharmacon, M-013167-00-0005) using Lipofectamine RNAiMAX (Invitrogen, 13778-150). For each reaction, 5 μl of Lipofectamine RNAiMAX was added to 150 μl of Opti-MEM Medium. Twenty nanomolar of siRNA was added to another tube with 150 μl of Opti-MEM Medium. Both were combined, vortexed, and incubated for 20 min at RT. Three hundred microliters of siRNA-Lipofectamine mix was added per well with 1.7 ml media. Twenty-four hours after transfection, cells were trypsinized and seeded onto a glass coverslip in 24-well plates to be fixed for imaging or onto six-well plates to be lysed for western blots the day after.

### smFISH probes

Custom single-molecule fluorescence *in situ* hybridization (smFISH) probes against WAC, NAA50 (ENST00000240922.7), PURB (ENST00000395699.3), and SLMO2 (ENST00000355937.8) were designed with Stellaris RNA FISH Probe Designer and labeled with Quasar 670 dye based on a protocol described in [37]. Oligo (dT)30-Cy3 probes were purchased from IDT. Single-molecule inexpensive fluorescence *in situ* hybridization (smiFISH) probes for RLuc were generated as described in [38] with minor adjustments. RLuc smiFISH probes were designed with the Biosearch Technologies Stellaris Probe Designer version 4.2. Both probes and the secondary FLAP-Y sequence were ordered from IDT. Primary probes were pooled in equimolar amounts to a final concentration of 100 μM total oligo.

### Sequential IF and FISH

Sequential immunofluorescence (IF) and smFISH/smiFISH on fixed U-2 OS cells were performed with homemade buffers [39] according to the manufacturer’s protocol: (https://biosearchassets.blob.core.windows.net/assets/bti_custom_stellaris_immunofluorescence_seq_protocol.pdf). Briefly, U-2 OS cells were seeded on sterilized coverslips in 24-well plates. At ∼80% confluency, media were exchanged 1 h before experimentation with fresh media. After stressing cells (see section “Stress conditions”), the media were aspirated and the cells were washed with 1× PBS pre-warmed to 37°C. The cells were fixed with 350 μl of 4% paraformaldehyde for 11 min at room temperature. After fixation, cells were washed three times with 1× PBS, permeabilized in 250 μl 0.1% Triton X-100 in 1× PBS for 5 min, and washed once with 1× PBS. For IF detection, coverslips were incubated in primary antibody for 1 h at room temperature. Coverslips were washed three times with 1× PBS. Then cells were incubated in secondary antibody for 1 h at room temperature. Again, coverslips were washed three times with 1× PBS. Subsequently, cells were fixed again with 350 μl of 4% paraformaldehyde for 11 min at room temperature and washed thrice with 1× PBS. Cells were then treated with smFISH Buffer A for 5 min. Coverslips were transferred to a humidifying chamber with smFISH probes and placed in the dark at 37°C for 16 h. Afterward, coverslips were placed in Buffer A for 30 min in the dark, replaced with fresh Buffer A for another 30 min in the dark, washed twice with Buffer B for 5 min each, and placed onto a slide with VECTASHIELD Antifade Mounting Medium with DAPI (Vector Labs, H-1200).

To maintain consistency, the same protocol was utilized in IF-only experiments; however, the portions of the protocol calling for smFISH were omitted. Coverslips were mounted with Prolong Glass Antifade Mountant with NucBlue Stain (Thermo Fisher Scientific, P36981). Antibodies used are listed in Supplementary Table S1.

### Microscopy

Fixed U-2 OS cells stained by IF and smFISH were imaged using the inverted Nikon Ti2 Eclipse spinning disk confocal microscope with either a 60× NA 1.27 water immersion objective or a 100× NA 1.45 oil immersion objective and a Hamamatsu ORCA Fusion BT sCMOS camera. At least five images were recorded at room temperature per biological replicate, with z-sections taken for each experiment. Every experiment was performed in three biological replicates, unless stated otherwise.

### Image analysis and quantification

Image processing was conducted using Fiji image processing package, with all shown images being the maximum intensity projection of a series of z-sections. Image segmentation masks, quantification of fluorescence intensities, SG counts, smFISH spots, and cell measurements were obtained using CellProfiler 4.2.5. During quantification of FISH spots, only cytoplasmic FISH spots were counted by segmenting out the cell cytoplasm using the PABPC1 and DAPI channels. Partition coefficients were determined by the number of FISH spots in stress granule/cytoplasm and analyzed using R-studio. To count cells with stress granule-like foci, images were blinded and manually examined. At least 50 cells were counted per biological replicate.

### Statistical analysis

Unpaired, two-sided *t*-test was performed on the mean of all biological replicates. In experiments with more than two groups, one-way ANOVA with Tukey’s multiple comparisons test were performed and the adjusted *P*-value were reported, unless stated otherwise. All statistical analysis was performed in GraphPad Prism 8.0.

## Results

### Generation of a luciferase reporter system to probe stress granule accumulation

**Figure 1.**
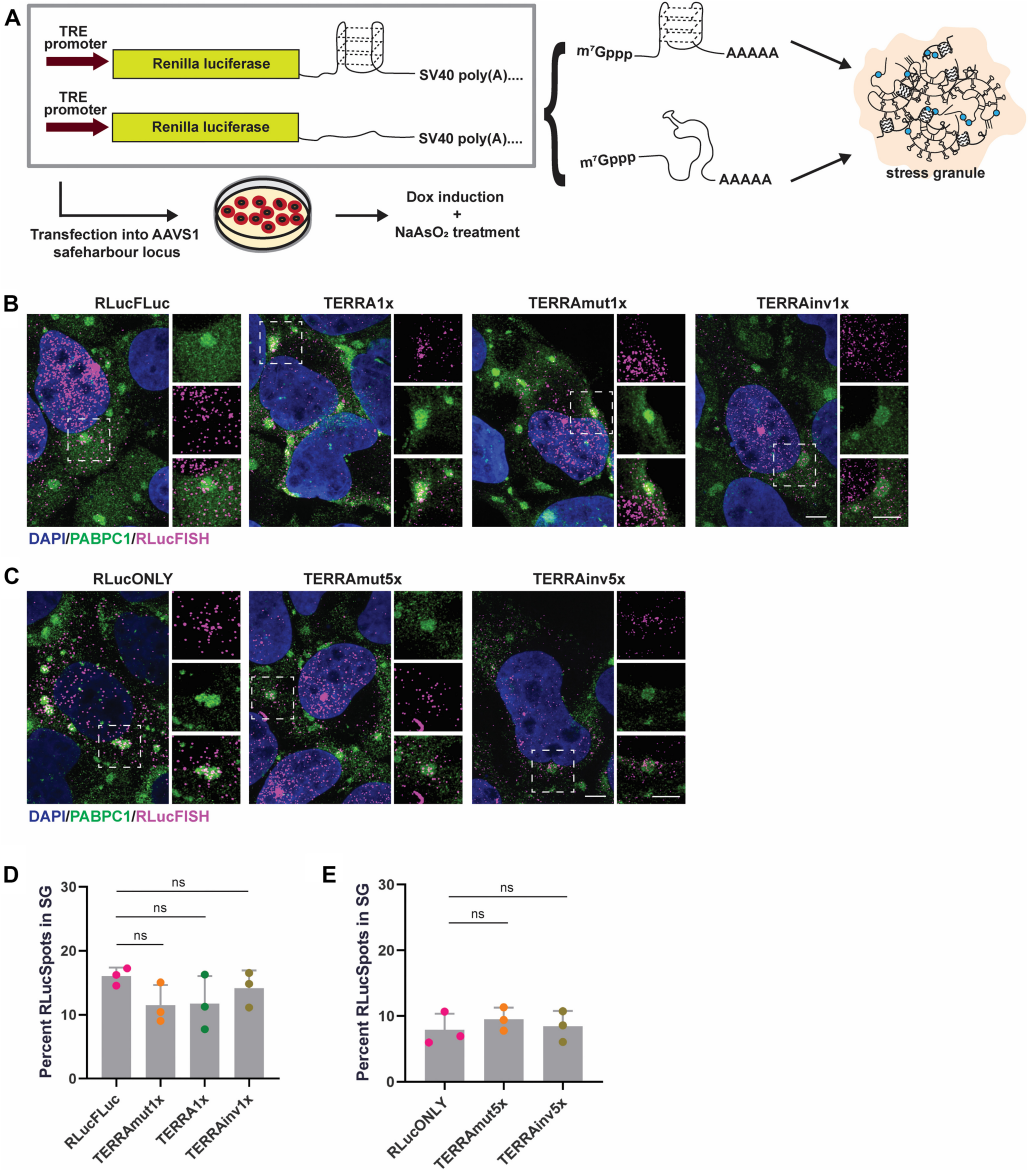
3′ UTR G-tracts do not alter mRNA abundance in stress granules in U-2 OS cells. (**A**) Schematic of rG4 reporter localization. To determine whether 3′ UTR G-tract mRNA is preferentially found in stress granules, G-tract sequences with quadruplex-forming potential or controls devoid of consecutive guanines are inserted into the 3′ UTR of RLuc. The reporter is tetracycline-inducible, where addition of doxycycline activates the TRE promoter to transcribe the downstream reporter mRNA. Reporter constructs are stably transfected into the *AAVS1* locus of U-2 OS cells. Blue circle: RNA binding protein. IF of DAPI, PABPC1, and RLuc RNA in U-2 OS cells stably expressing (**B**) 1× reporter constructs and (**C**) 5× reporter constructs treated with 500 μM NaAsO_2_ for 60 min. Scale bar = 5 μm. (**D**, **E**) Quantification of RLuc RNA FISH spots in stress granules as in panels (B) and (C), respectively. Data analyzed with one-way ANOVA, corrected with Tukey’s multiple comparisons test, and represented as mean ± SD. ns = non-significant, *P* > .05. Three biological replicates were quantified. Reporter sequences in Supplementary Table S1.

To test the effect of G-tract sequences on mRNA localization, we created reporter mRNAs with and without G-tracts inserted into the 3′ UTR and then examined the abundance of the reporter in stress granules. The luciferase reporters had either predicted rG4-forming sequences or non-rG4-forming sequences in 1× or 5× copy numbers in their 3′ UTRs, where 1× signifies four sequence repeats potentially forming one rG4 structure (Fig. 1A and Supplementary Table S1). Reporter constructs included a tetracycline-on expression system so that the RNAs should only be expressed in the presence of doxycycline (abbreviated as Dox in figures). These constructs were targeted to the *AAVS1* safe harbor locus for integration and stable expression following transfection in WT U-2 OS cells using CRISPR/Cas9 genome editing and a donor template DNA. However, it should be noted that what fraction, if any, of our reporter RNAs form stable rG4 structures in cells is undetermined, as cellular conditions may favor unfolding by RNA helicases [40, 41] and/or promote the formation of alternative, competing secondary structures.

We introduced a specific set of sequence motifs, computationally predicted to adopt rG4 conformations, into the 3′ UTR. One of the best understood non-coding RNAs that forms rG4s is the telomeric repeat-containing RNA (TERRA) [42–44]. In parallel, we also generated a construct containing an inverted TERRA sequence (Supplementary Table S1), which would also be predicted to form an rG4 (referred to as TERRAinv). Using circular dichroism, we confirmed that the 24-mer TERRAinv formed similar rG4 structures to that of TERRA in the presence of K^+^*in vitro* with a distinguishing positive peak at 210 nm (Supplementary Fig. S1A), while rG4 signatures were abolished in Li^+^ solution (Supplementary Fig. S1B). As a control, we generated a construct that contains the same G-content as the rG4 motifs but lacks consecutive guanine bases. This construct, referred to as TERRAmut, has reduced rG4-forming capability, as confirmed via circular dichroism, where it showed a negative peak at 210 nm that is indicative of A-form RNA rather than rG4 [45] (Supplementary Fig. S1A and B).

The functional effects of rG4 sequence motifs are highly dependent on their transcriptome context, including the nature of their flanking sequences. To better mimic the context of an endogenous mRNA, we included 50-bp and 61-bp sequences derived from the regions flanking the rG4 sequence motif in the endogenous APP transcript [46], placing them upstream and downstream of 1 × 3′ UTR G-tract sequences, respectively (Supplementary Table S1). In our 5× constructs, we included a 23-bp spacer sequence between adjacent sets of G-tract repeats (Supplementary Table S1). To minimize the exclusion of G-tract motifs by recombination, each spacer sequence was slightly varied.

We validated that all reporter constructs were successfully integrated into the *AAVS1* locus using primers flanking RLuc sequences (Supplementary Fig. S1C). We also verified in RLucFLuc U-2 OS cells that RLuc and firefly luciferase proteins were expressed only upon doxycycline induction and were enzymatically active, demonstrating the mRNAs were fully functional (Supplementary Fig. S1D and E).

### Potential rG4 motifs in 3′ UTRs do not alter mRNA accumulation in stress granules

To determine whether G-tracts in 3′ UTRs affect mRNA accumulation in stress granules, we examined the localization of our reporter mRNAs using IF. We induced expression of the reporter constructs containing G-tracts with a single rG4 motif-forming potential by treating cells with doxycycline for 48 h, followed by NaAsO_2_-induced oxidative stress for 1 h. We then imaged the localization of RLuc reporter mRNAs in the cell with smiFISH, which uses unlabeled gene-specific probes with a FLAP sequence that is subsequently recognized by the fluorescently labeled secondary detector probes [38, 47]. To confirm the validity of the smiFISH signals, we also stained cells for FLuc and RLuc proteins. The cells with RLucFISH signals were also positive for either FLuc (Fig. 1B and Supplementary Fig. S2A, C and E) or RLuc (Fig. 1C and Supplementary Fig. S2B).

The fraction of the reporter mRNAs localized to stress granules was then determined by quantification of the number of smiFISH spots overlapping with PABPC1 + stress granules, compared to the total number of cytoplasmic smiFISH spots (see the “Materials and methods” section). We observed no statistical difference in the fraction of reporter mRNAs accumulating in stress granules with or without the insertion of 3′ UTR G-tracts. Specifically, 11.8% ± 4.3% and 14.2% ± 2.8% of the TERRA and TERRAinv reporter mRNAs, respectively, while 11.5% ± 3.2% of the TERRAmut mRNAs accumulated in stress granules (Fig 1B and D). Thus, these experiments did not support the preferential enrichment of rG4 sequence motifs in stress granules.

In addition to our artificial 3′ UTR TERRA sequences, we also inserted the 3′ UTR rG4 sequence motifs from the endogenous mRNA, APP, into our reporter system (Supplementary Table S1). The APP 3′ UTR rG4 sequence has been previously validated to form rG4 structures [46]. As controls, we generated two previously reported APP mutant constructs that have decreasing rG4-forming potential [46]. APPG2 has some of the guanines mutated so that it can form a two-tetrad rG4 structure but is less stable than the canonical three-tetrad rG4. On the other hand, consecutive guanines are fully abolished in APPmut, making it incapable of forming rG4 structures.

Similar to the 1× TERRA constructs, we did not observe differences in reporter mRNA accumulation in stress granules in the presence of 3′ UTR APP rG4 sequence motifs (Supplementary Fig. S2C and D). Thus, having 3′ UTR G-tracts that are predicted to form a single rG4 in an mRNA did not alter its accumulation in stress granules in our U-2 OS cells.

The assembly of macromolecules into condensates is generally proportional to the number of interaction sites [11]. Indeed, we have previously demonstrated that mRNA localization into stress granules can be enhanced by increasing protein binding sites on the mRNA in a dose-dependent manner [48]. Given this, we considered the possibility that the contribution of a single rG4 sequence motif in an mRNA to stress granule accumulation might be very small and difficult to detect. We therefore tested reporter constructs harboring five repeats of the putative rG4s in the 3′ UTR to determine whether adding more rG4 sequence motifs could alter mRNA localization to stress granules. However, having five putative rG4-forming sequence motifs at the 3′ UTR also did not increase reporter mRNA accumulation in stress granules (Fig. 1C and E). Taken together, this suggests that the presence of one or more 3′ UTR rG4 sequence motifs does not affect mRNA recruitment into stress granules in U-2 OS cells.

### mRNA length predicts stress granule occupancy better than rG4 motifs

DHX36 is a member of the DEAH-box helicase family that has been shown to tightly bind rG4s and unwind them *in vitro* [26, 28, 49]. Given the rG4 helicase activity of DHX36, we considered the possibility that it is unwinding rG4 structures in cytoplasmic mRNAs and thereby preventing rG4s from promoting mRNA accumulation in stress granules. To test this model, we first investigated DHX36 localization in U-2 OS WT cells using IF. Consistent with previous results, DHX36 is mostly cytoplasmic, and upon arsenite stress, DHX36 is enriched in stress granules as indicated by colocalization with PABPC1; therefore, DHX36 could affect RNA structures within stress granules (Supplementary Fig. S3C) [23, 30, 31].

We examined whether DHX36 affected the stress granule accumulation of endogenous mRNAs with rG4-forming potential. Previous work suggested that DHX36 mRNA targets based on PAR-CLIP were enriched in stress granules [23]. However, length is also a key determinant for mRNA enrichment to stress granules [5] due to increased multivalency [48]. Here, we cross-referenced the DHX36-E335A PAR-CLIP data [23] with the stress granule transcriptome [5] and binned DHX36 target mRNAs in accordance with the number of cross-linked reads per target mRNA normalized by overall mRNA abundance (normalized cross-linked reads per million, NXPM) obtained by DHX36-E335A PAR-CLIP [23]. The DHX36-E335A mutant lacks helicase catalytic activity due to its inability to hydrolyze ATP [31]. This locks DHX36 in an RNA-bound conformation that enables efficient pull-down of its target mRNAs [50].

**Figure 2.**
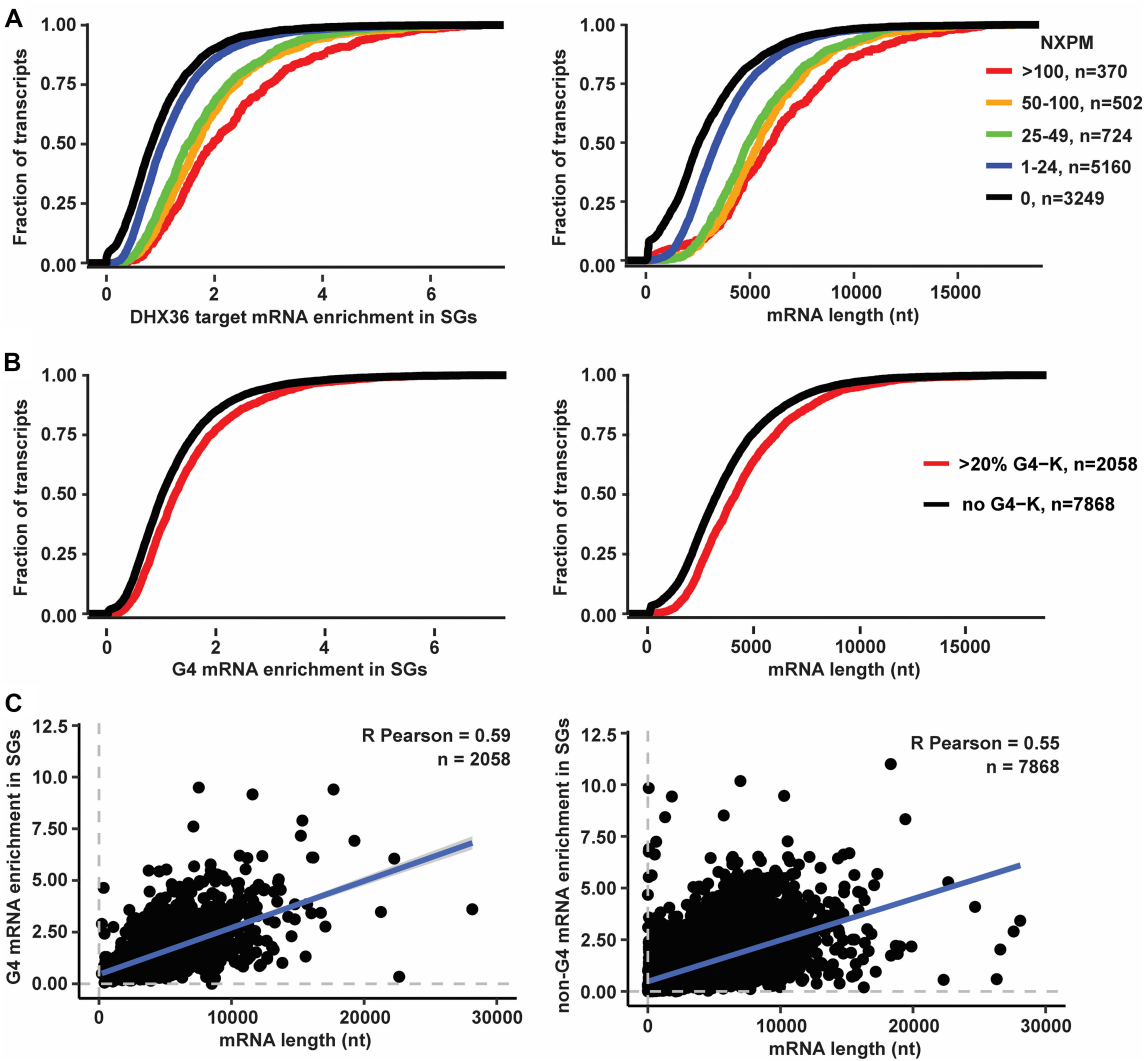
Enrichment of rG4 motifs in stress granule-localized mRNA appears to be a consequence of mRNA length. (**A**) Cumulative distribution functions showing (left) enrichment in stress granules of DHX36 target mRNAs identified in DHX36-E335A PAR-CLIP [23] compared to non-target mRNAs and (right) length distribution of DHX36 target mRNAs compared to non-target mRNAs. DHX36 target mRNAs are binned in accordance to the number of normalized cross-linked reads per million (NXPM), as described in [23]. (**B**) Cumulative distribution functions showing (left) enrichment of mRNA transcripts harboring G4 motifs in K^+^ identified previously in [21] compared to non-rG4-harboring transcripts and (right) length distribution of G4-harboring mRNA transcripts compared to non-rG4-harboring transcripts. (**C**) Scatterplot showing correlation between stress granule enrichment of G4 mRNA (left) or non-G4 mRNA (right), as determined in [21], and mRNA length. Data on mRNA enrichment in stress granules is obtained from [5].

Our analyses suggest that the enrichment of DHX36 targets in stress granules is likely a result of longer mRNAs having an enrichment of DHX36 targets. Notably, we observed that while RNAs with increasing DHX36-binding sites were more likely to be enriched in stress granules (Fig. 2A, left), these RNAs also tended to be longer (Fig. 2A, right). A similar result was observed when integrating data from transcripts harboring canonical rG4 motifs [21] (Fig. 2B and C). In this second dataset, the presence of rG4s was defined by a reverse transcriptase stalling (RTS) value >0.2 [21], which means that >20% of the reads containing the rG4 motif showed stalling. Thus, a value of 0.2 suggests that some type of RNA structure, possibly an rG4, was present in 20% of the population of a particular transcript. The comparison of transcripts without rG4s and transcripts with >20% rG4 showed that transcripts harboring rG4s tended to be longer (Fig. 2B, right), which again correlated with stress granule enrichment (Fig. 2B, left). As an orthogonal approach, we compared mRNAs containing putative rG4 motifs with those lacking such motifs to examine the relationship between stress granule enrichment and DHX36 mRNA target length. Both groups exhibited a positive correlation between stress granule enrichment and mRNA transcript length, regardless of their RTS values (Fig. 2C). Thus, our analyses are consistent with mRNA length, rather than the presence of rG4 motif in an mRNA, dictating an mRNA’s enrichment to stress granules. It should be noted that one limitation of these analyses is that we are comparing datasets obtained in different cell lines.

### DHX36 depletion does not cause accumulation of endogenous RNAs with rG4 motifs in stress granules

We then experimentally examined whether the loss of DHX36 increased the accumulation of DHX36 target mRNAs with rG4 sequence motifs in stress granules. We used Cas9 targeting the *DHX36* gene with two guide RNAs (Supplementary Table S1) to obtain cell lines with reduced DHX36 protein levels. We obtained colonies with stably reduced DHX36 expression [hereafter DHX36hm (for hypomorph)] in U-2 OS cells as shown by western blotting (Supplementary Fig. S3A and B left). No colonies were found with a complete loss of DHX36, arguing that DHX36 is essential in U-2 OS cells. As reported in HEK293 cells [23], DHX36hm cells displayed slower growth rate and different morphology due to inefficient spreading in the culture dish (not shown).

Since DHX36 KO has been reported to produce spontaneous stress granules by activating protein kinase R (PKR) in the integrated stress response pathway [23], we examined stress granule formation and PKR phosphorylation in our stable cell lines without stress induction. We did not observe spontaneous stress granules in our DHX36hm cell lines in unstressed conditions, as indicated by PABPC1 IF (Supplementary Fig. S3C). Western blot analyses further corroborated that in the absence of arsenite stress, the levels of phospho-PKR and phospho-eIF2α in DHX36hm did not increase (Supplementary Fig. S3D–F). It should be noted that we might not observe the activation of PKR and spontaneous stress granule formation because we are working with a hypomorphic DHX36 cell line, because of better stress recovery in stable cell lines, or because of differences between U-2 OS and HEK293 cells.

Using these stable DHX36hm cell lines, we performed smFISH and quantified the fraction enrichment of DHX36 target mRNAs in stress granules. In this experiment, we designed and tested FISH probes for the DHX36 targets WAC, NAA50, PURB, and SLMO2, which were among the top 100 DHX36 targets in the PAR-CLIP analysis [23], and their rG4-forming potential is supported by multiple orthogonal methods (Table 1). These four DHX36 target mRNAs also harbor rG4 sequence motifs at different regions within the transcript (Table 1), allowing for an unbiased assessment of the impact of rG4 sequence motifs on accumulation of mRNAs in stress granules. This experiment provided two observations, revealing how DHX36 affects mRNA levels and localization.

**Table 1.** Regions within the mRNA transcript that harbor putative rG4s

Gene symbol	Regions of experimentally determined rG4s		Regions of predicted rG4s by various prediction tools		
	RT-stop profiling	rG4-seq	QGRSmapper	pqsfinder	G4Hunter
**WAC**	3′ UTR	-	5′ UTR, 3′ UTR	5′ UTR, 3′ UTR	5′ UTR, 3′ UTR
**NAA50**	5′ UTR	5′ UTR, 3′ UTR	5′ UTR	5′ UTR, 3′ UTR	3′ UTR
**PURB**	3′ UTR	CDS, 3′ UTR	CDS	5′ UTR, CDS, 3′ UTR	3′ UTR
**SLMO2/PRELID3B**	3′ UTR	5′ UTR, 3′ UTR	3′ UTR	3′ UTR	-

*Data obtained from QUADRatlas [54].

**Figure 3.**
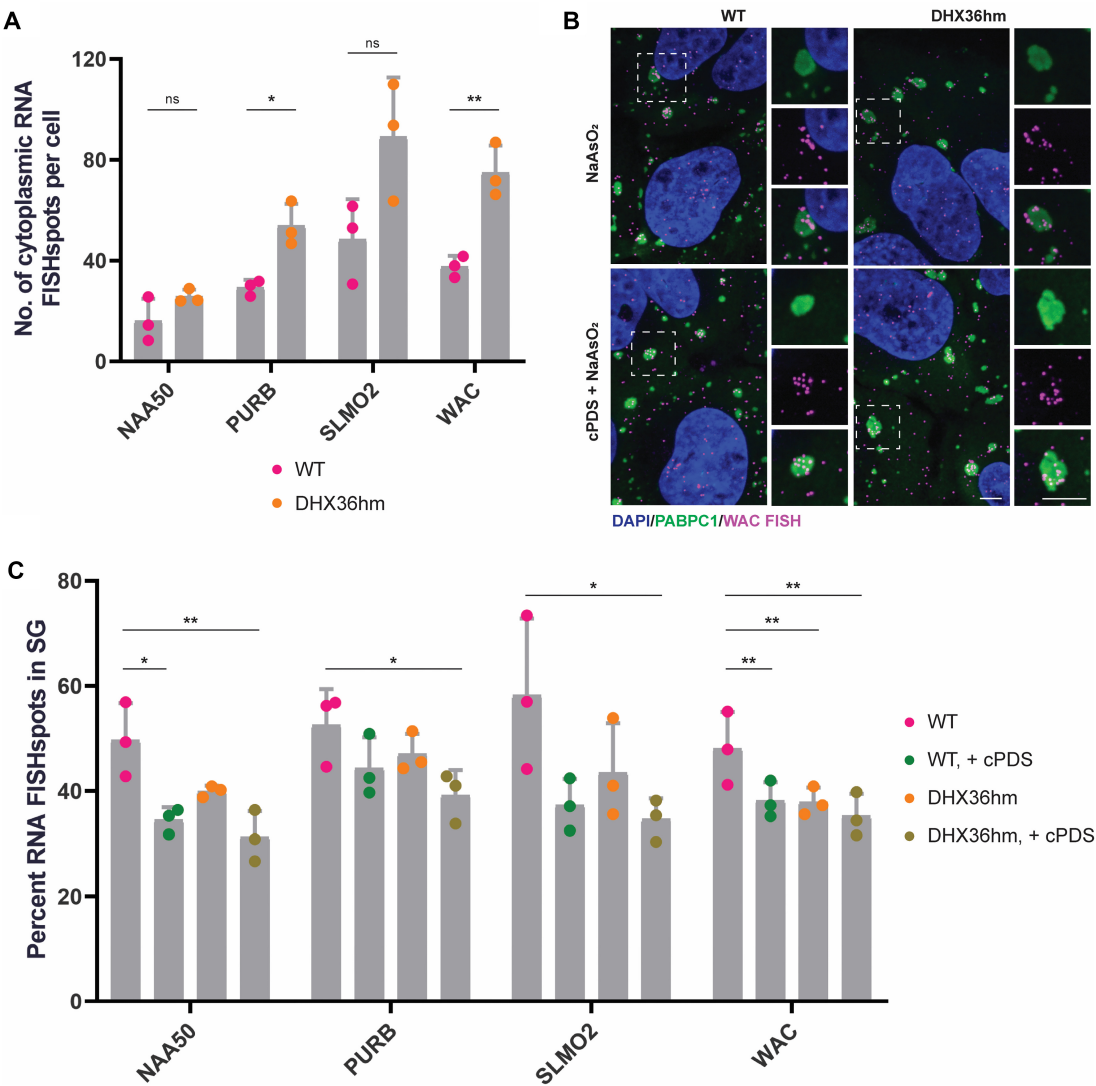
Stress granule localization of endogenous mRNAs with rG4 sequence motifs does not increase with DHX36 depletion and cPDS treatment. (**A**) Quantification of NAA50, PURB, SLMO2, and WAC RNA FISH spots per cell in cells treated with 500 μM NaAsO_2_ for 60 min. Data were analyzed with two-tailed unpaired *t*-test and represented as mean ± SD. (*P = .0101, **P = .0049). ns = non-significant, *P* > .05. Three biological replicates were quantified. (**B**) IF of DAPI, PABPC1, and WAC RNA in WT and stable DHX36hm U-2 OS cells treated with either 500 μM NaAsO_2_ for 60 min or with 2 μM cPDS for 24 h and 500 μM NaAsO_2_ for 60 min. Scale bar = 5 μm. (**C**) Quantification of percent NAA50, PURB, SLMO2, and WAC RNA localized in stress granules. Cells were treated with 500 μM NaAsO_2_ for 60 min or with 2 μM cPDS for 24 h and 500 μM NaAsO_2_ for 60 min. Data were analyzed with RM one-way ANOVA, corrected with Dunnett’s multiple comparisons test, and represented as mean ± SD. (NAA50: *P-adj = .0224, **P-adj = .0095. PURB: *P-adj = .0194. SLMO2: *P-adj = .0330. WAC: **P-adj = .0090, **P-adj = .0080, **P-adj = .0025). ns = non-significant, *P* > .05. Three biological replicates were quantified.

First, in U-2 OS DHX36hm cells, we saw an increase in the number of cytoplasmic smFISH spots for candidate DHX36 target mRNAs (Fig. 3A). This increase is expected based on the decreased mRNA decay previously observed for these mRNAs in DHX36 KO HEK293 cells [23].

Second, we observed that depletion of DHX36 did not increase the fraction of candidate DHX36 target mRNAs localized in stress granules (Fig. 3B and C and Supplementary Fig. S4A–C). This argues that DHX36 does not play a major role in modulating the accumulation of these endogenous mRNAs in stress granules.

To further push the equilibrium toward RNA structure formation in cells, we examined the localization of DHX36 target mRNAs in the presence of carboxypyridostatin (cPDS), a small molecule that preferentially binds to and stabilizes rG4 structures [43]. However, following cPDS treatment, we did not observe any increase in the abundance of DHX36 target mRNAs within stress granules (Fig. 3B and C). In fact, if anything, there was a trend for these mRNAs to be excluded from stress granules upon cPDS treatment (Fig. 3C). These observations support the hypothesis that rG4 sequence motifs within mRNAs do not enhance their recruitment into stress granules.

The formation of RNA secondary structures can act as kinetic barrier to ribosome progression, leading to ribosome stalling on mRNAs [51]. Stalled ribosomes will impede the recruitment of these transcripts to stress granules, as ribosome occupancy is generally antagonistic to stress granule localization [52]. To ensure that DHX36 depletion or cPDS treatment did not promote ribosome stalling, we treated cells with puromycin, which induces premature chain termination and disassembles ribosomes from mRNAs [53], thereby releasing any potentially stalled ribosomes. In both WT and DHX36hm cells, puromycin cotreatment with cPDS did not increase the enrichment of DHX36 target mRNAs in stress granules (Supplementary Fig. S4D). This demonstrates that the lack of stress granule enrichment in DHX36 target mRNAs is not due to ribosome stalling.

Taken together, our results suggest that rG4 sequence motifs within the mRNAs examined do not affect their enrichment in stress granules and that the accumulation of endogenous mRNAs with rG4 motifs in stress granules is independent of DHX36 and cPDS treatment.

### DHX36 depletion facilitates stress granule assembly and slows disassembly

DDX6 and eIF4A are DEAD-box RNA helicases that function as “RNA chaperones” to limit stress granule assembly [32, 34]. We considered the possibility that DHX36 might function as a part of this RNA chaperone network by altering the protein composition of mRNPs and/or by altering RNA structure.

**Figure 4.**
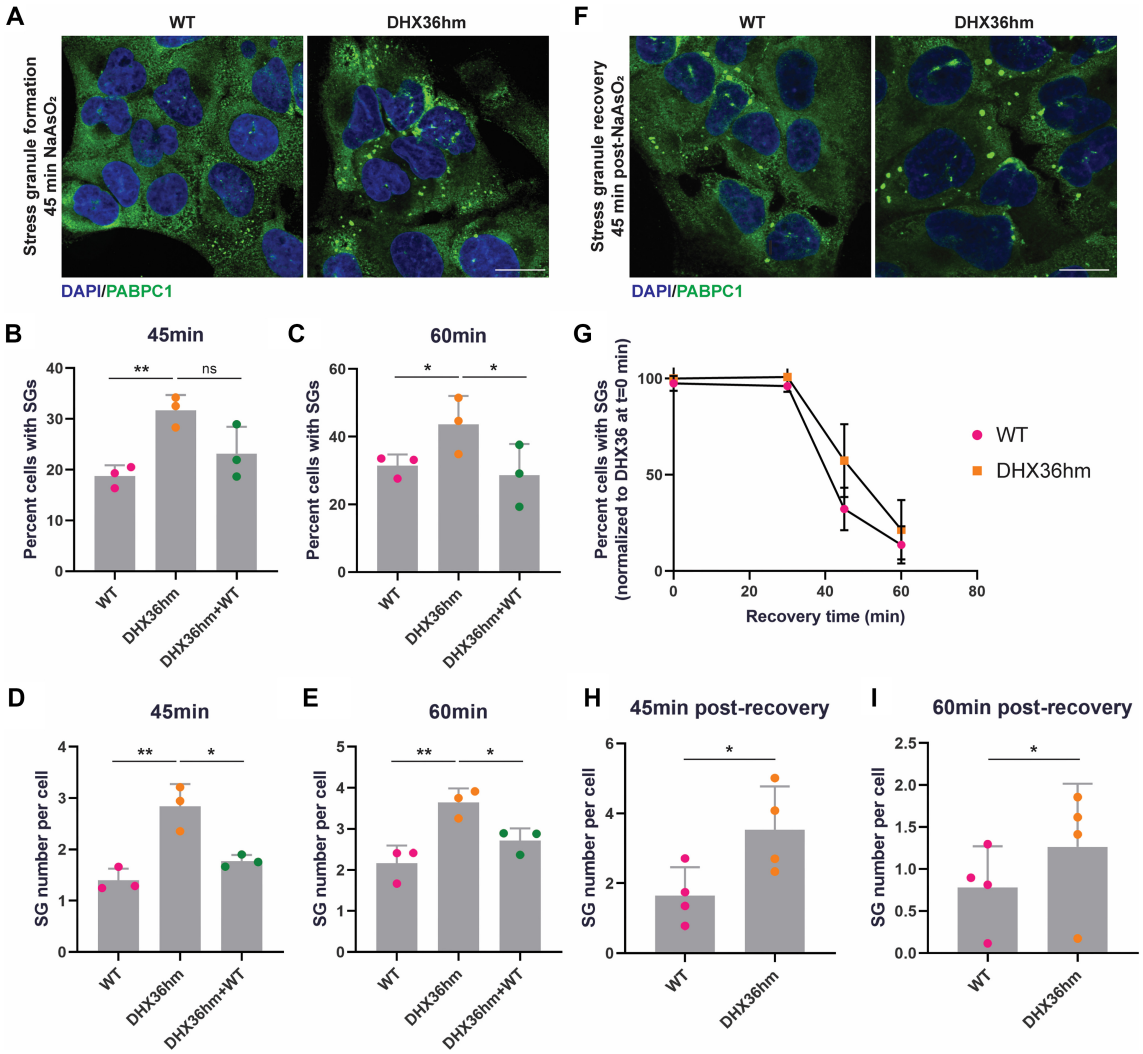
DHX36 depletion facilitates stress granule assembly and slows disassembly. (**A**) IF of DAPI and PABPC1 in WT and stable DHX36hm U-2 OS cells treated with 100 μM NaAsO_2_ for 45 min. Scale bar = 20 μm. Quantification of percent WT, DHX36hm, and DHX36hm + WT U-2 OS cells containing 2 or more stress granules after treating with 100 μM NaAsO_2_ for (**B**) 45 min and (**C**) 60 min. Data were analyzed with one-way ANOVA, corrected with Tukey’s multiple comparisons test, and represented as mean ± SD (*t* = 45 min: **P-adj = .0035. *t* = 60 min: *P-adj = .0477 [WT versus DHX36hm], *P-adj = .0182 [DHX36hm versus DHX36hm + WT]). Three biological replicates were quantified. Quantification of stress granule number per cell in WT, DHX36hm, and DHX36hm + WT cells treated with 100 μM NaAsO_2_ for (**D**) 45 min and (**E**) 60 min. Data were analyzed with one-way ANOVA, corrected with Tukey’s multiple comparisons test, and represented as mean ± SD (*t* = 45 min: **P-adj = .0023, *P-adj = .0103; *t* = 60 min: **P-adj = .0058, *P-adj = .0459). Three biological replicates were quantified. (**F**) IF of DAPI and PABPC1 in WT and stable DHX36hm U-2 OS cells treated with 300 μM NaAsO_2_ for 30 min and recovered in DMEM for 45 min. Scale bar = 20 μm. (**G**) Quantification of WT and DHX36hm U-2 OS cells containing 2 or more stress granules after treating with 300 μM NaAsO_2_ for 30 min and allowed to recover in DMEM for *t* = 0, 30, 45, and 60 min. Data were analyzed with two-tailed unpaired *t*-test and represented as mean ± SD. Three biological replicates were quantified. Quantification of stress granule number per cell after recovering from stress for (**H**) 45 min and (**I**) 60 min. Data were analyzed with two-tailed paired *t*-test and represented as mean ± SD. (*t* = 45 min: *P = .0174. *t* = 60 min: *P = .0451). Four biological replicates were quantified.

To understand whether DHX36 can affect stress granule dynamics, we first examined changes in stress granule assembly and disassembly kinetics when DHX36 expression was dampened. We assessed stress granule assembly kinetics by exposing cells to low amounts of NaAsO_2_ (100 μM) to allow sensitive measurements of stress granule formation. We measured the rate of stress granule formation over time using IF against PABPC1. We assessed the percent of cells that formed stress granules on a population level and observed that stress granules reproducibly formed at a faster rate in DHX36hm cells compared to WT cells (Fig. 4A–C). This difference was particularly appreciable at 45 min and 60 min post-NaAsO_2_ treatment (Fig. 4B and C). On average, cells also produced more stress granules at 45 min and 60 min post-NaAsO_2_ treatment when DHX36 was depleted (Fig. 4D and E).

We further confirmed that this was a direct effect of DHX36 by a rescue experiment (Supplementary Fig. S5A). Overexpression of FLAG-DHX36 WT in DHX36hm cells led to a reversal in the rate of stress granule formation (Fig. 4B and C) and number of stress granules per cell (Fig. 4D and E), similar to that of WT levels. These observations demonstrate a role for DHX36 in enhancing stress granule nucleation and align with earlier work that implicated DHX36 in stress granule assembly [27].

We also examined if DHX36 affects stress granule disassembly after stress is removed. We treated cells with 300 μM NaAsO_2_ for 1 h to ensure that stress granules were formed in all cells before removing the stress. Following arsenite removal, we observed that stress granules in DHX36hm cells persisted longer than in WT cells (Fig. 4F and G). Although not statistically significant, the trend of DHX36hm cells displaying slower stress granule dissolution was consistent in our replicates at both 45 min and 60 min post-recovery. In addition, DHX36hm cells exhibited a higher number of stress granules per cell compared to WT at both 45 min and 60 min post-recovery (Fig. 4H and I), suggesting that DHX36 repression impaired stress granule disassembly in cells.

Taken together, these two observations suggest that DHX36 can function to limit stress granule formation and promote disassembly.

### DHX36 depletion restores stress granule-like foci in G3BP1/2 dKO cells

In an orthogonal approach to ascertain whether DHX36 can limit stress granule assembly, we performed siRNA and CRISPR knockdown of DHX36 in cell lines lacking G3BP1 and G3BP2 (Supplementary Fig. S3A and B). G3BP1 and its paralog G3BP2 are major assembly factors of stress granules. Stress granule formation is compromised in cells lacking these two proteins due to impaired G3BP1/2-mediated protein–protein and/or protein–RNA interactions [8]. These cell lines therefore provide a sensitive assay to detect proteins that restrict stress granule formation via limiting RNA–RNA or RNA–protein interactions [32]. If a protein such as the DEAD-box helicase eIF4A or DDX6 functions to limit stress granule formation through restricting RNA-mediated interactions, one can observe a restoration of small stress granule-like foci in G3BP1/2 dKO cells as seen when eIF4A or DDX6 is functionally inhibited [32, 34].

**Figure 5.**
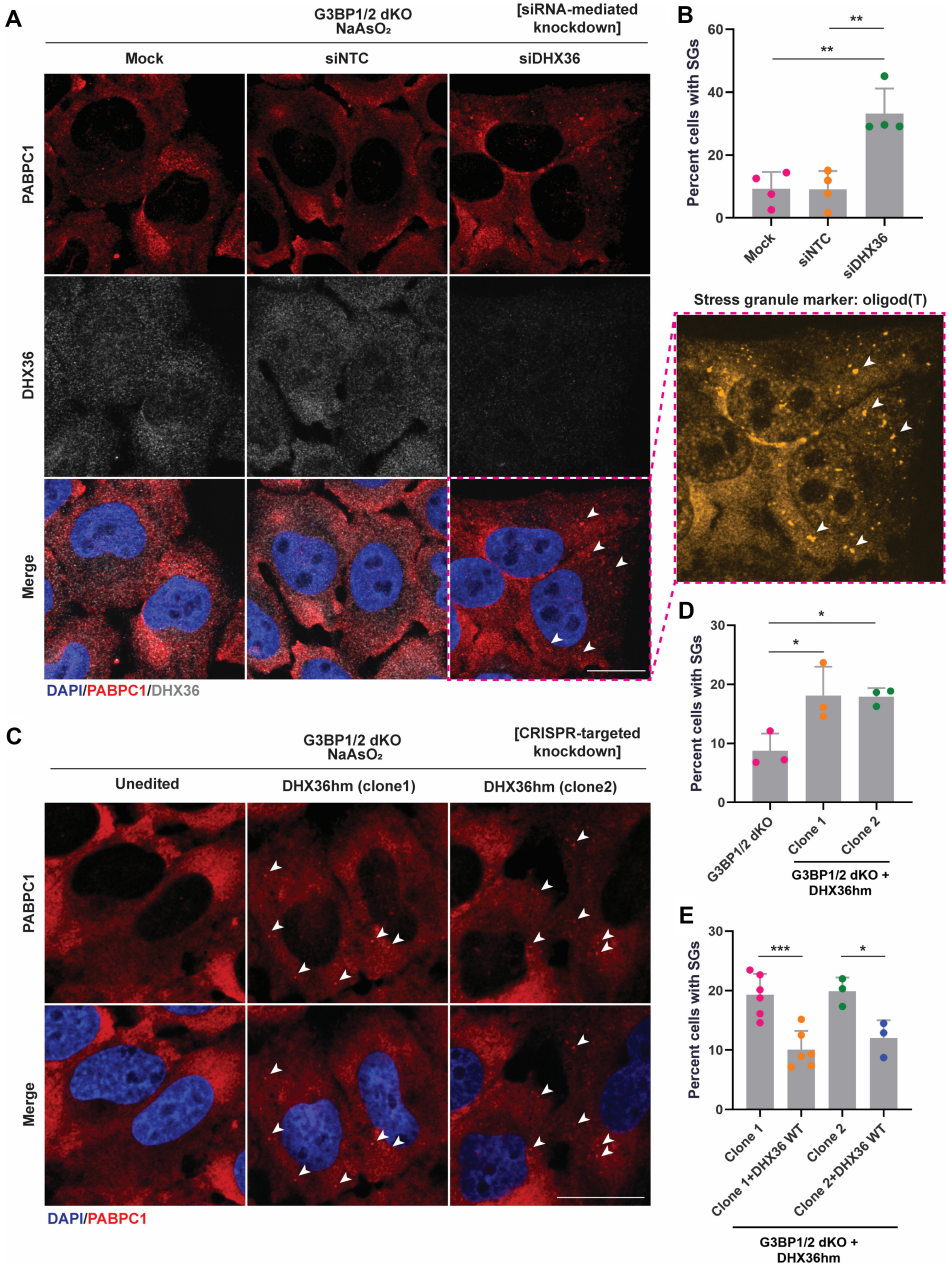
DHX36 depletion restores stress granule-like foci in G3BP1/2 dKO cells. (**A**) IF of DAPI, PABPC1, oligo (dT) RNA, and DHX36 in G3BP1/2 dKO U-2 OS cells transfected with either siNTC (non-targeting control) or siDHX36. Forty-eight hours post-transfection, cells were treated with 500 μM NaAsO_2_ for 60 min. Arrows: representative stress granule foci. Scale bar = 20 μm. (**B**) Quantification of cells with stress granules as shown in panel (A). Data were analyzed with one-way ANOVA, corrected with Tukey’s multiple comparisons test, and represented as mean ± SD. (**P-adj = .0014 [Mock versus siDHX36], **P-adj = .0014 [siNTC versus siDHX36]). Four biological replicates were quantified. (**C**) IF of DAPI and PABPC1 in G3BP1/2 dKO and G3BP1/2 dKO + DHX36hm U-2 OS cells treated with 500 μM NaAsO_2_ for 60 min. Arrows: representative stress granule foci. Scale bar = 20 μm. (**D**) Quantification of cells with stress granules as shown in panel (C). Data were analyzed with one-way ANOVA, corrected with Tukey’s multiple comparisons test, and represented as mean ± SD. (*P-adj = .033 [G3BP1/2 dKO versus Clone 1], *P-adj = .036 [G3BP1/2 dKO versus Clone 2]). Three biological replicates were quantified. (**E**) Quantification of cells with stress granules in two independent clones of G3BP1/2 dKO + DHX36hm U-2 OS cells rescued with FLAG-DHX36 WT. Data were analyzed with one-way ANOVA, corrected with Tukey’s multiple comparisons test, and represented as mean ± SD. (*P-adj = .039, **P-adj = .0009). Six biological replicates were quantified for Clone 1, and three biological replicates were quantified for Clone 2.

Strikingly, we observed a rescue of stress granule-like foci in 31.7% ± 12.5% of G3BP1/2 dKO cells with a transient knockdown of DHX36 using siRNAs (Fig. 5A and B). In comparison, stress granules were observed in 8.5% ± 3.3% of cells with the non-targeting control, as visualized by PABPC1 staining (Fig. 5A and B) and oligo(dT) RNA (Fig. 5A, right). Similarly, when we stably knocked down DHX36 in G3BP1/2 dKO cells with a Cas9 targeting strategy, we also observed a rescue of stress granule-like foci in 18.1% ± 4.9% and 17.9% ± 1.4% of cells in two independent knockdown clones of DHX36 compared to that 8.7% ± 3.0% in G3BP1/2 dKO cells (Fig. 5C and D).

To validate that this restoration of stress granule-like foci was due to decreased DHX36 function, we performed a rescue experiment where we transduced FLAG-DHX36 WT into the G3BP1/2 dKO + DHX36hm cell lines and confirmed DHX36 expression via immunoblot (Supplementary Fig. S5B). We showed that restoring DHX36 levels reversed the formation of stress granule-like foci in cells to 10.1% ± 3.1% and 12.0% ± 3.0% in two independent rescue cell lines (Fig. 5E and Supplementary Fig. S5C).

These observations suggest that DHX36 plays a role in limiting RNA condensation in stress granules. The simplest model is that DHX36 disrupts intermolecular RNA–RNA interactions that promote mRNA condensation. This effect could stem from DHX36 either limiting intermolecular *trans* rG4s or, more generally, inhibiting other types of non-rG4 intermolecular RNA–RNA interactions. Interestingly, we observed that addition of the rG4-stabilizing compound, cPDS, led to a three-fold increase in the number of G3BP1/2 dKO cells with stress granules, but had no effect on the number of cells with stress granules when DHX36 was knocked down (Supplementary Fig. S5D). This supports the idea that enhancing rG4 structure formation, either by stabilizing rG4s with cPDS or by depleting DHX36, can promote stress granule assembly.

## Discussion

Herein, we present several observations arguing that G-tracts in *cis* do not affect mRNA accumulation into arsenite-induced stress granules in U-2 OS cells. First, on the single mRNA level, we examined stress granule enrichment with both reporter and endogenous mRNAs that contained putative rG4-forming sequence motifs. The fraction of smFISH spots localized in stress granules compared to that in the cytoplasm was independent of having G-tracts within the mRNA (Fig. 1). Second, we found no correlation between having rG4 sequence motifs in an mRNA and its stress granule accumulation when we controlled for mRNA length (Fig. 2). Third, when we further pushed the cellular equilibrium to favor rG4 structure formation by knocking down the DHX36 helicase and/or adding the rG4-stabilizing ligand, cPDS, we observed no effect on stress granule accumulation of endogenous mRNAs with putative rG4 sequence motifs (Fig. 3). Although the idea that G-tract sequences facilitate the sequestration of mRNAs in stress granules through recognition by RNA-binding proteins such as G3BP has been much anticipated, our current observations challenge the generality of this expectation.

We consider two reasons why G-tracts that are putative rG4-forming sequences might be insufficient to alter mRNA localization in stress granules.

First, the effect of rG4 sequence motifs could be small and therefore be masked by other RNA features. This view is based on the fact that rG4 sequence motifs represent a small proportion of an mRNA, and their effect, if any, could be masked by the rest of the mRNA. Compared to the length of a typical mRNA of 2 kb, each rG4 motif (a canonical rG4 motif is defined by G_≥2_X_1–7_G_≥2_X_1–7_G_≥2_X_1–7_G_≥2_, where X is any other nucleotide) makes up only 0.8%–2% of the entire mRNA. For a stress granule-enriched mRNA with an average length of 7.1 kb [5], this percentage is even lower. However, in our reporter mRNAs with five repeats of the G-tract sequence, where the proportion of potential rG4-forming region in the mRNA increased to around 15%, there remained no change in mRNA accumulation (Fig. 1C and E). While we do not rule out the possibility that rG4 motifs can have as a small effect, we posit that the contribution of these motifs to an mRNA’s stress granule accumulation is minimal compared with other features of the mRNA, such as interactions with RNA-binding proteins and mRNA length. Moreover, rG4 structure formation may be outcompeted by alternative RNA secondary structures such as hairpins or internal loops.

A second issue is that at any given moment the majority of rG4s is likely to exist in an unfolded state *in cellulo* and therefore would not impact stress granule accumulation. Guo and Bartel [40] failed to detect rG4 structures in human cells and suggested that the dynamic formation of rG4s is regulated by RNA-binding proteins, particularly RNA helicases [40, 41]. On the other hand, Kharel *et al.* provided evidence suggesting that rG4 structures may become more prevalent during nutrient deprivation [55]. Therefore, while our findings do not support the role of rG4 sequence motifs in driving mRNA accumulation in stress granules under the conditions tested, we do not rule out the possibility that, in certain transcripts and under specific cellular states, the folding landscape of RNA could be altered and rG4 structures may have a more pronounced effect in modulating stress granule dynamics. However, it is important to note that under such conditions, the overall cellular homeostasis is remodeled, and starvation-induced reductions in cellular ATP levels could impair the activity of many RNA helicases.

In this study, we created a DHX36 hypomorph cell line with reduced DHX36 expression. We show that even when DHX36 levels are repressed, and RNA abundance is increased as shown previously [23, 56], the accumulation of endogenous rG4-containing mRNAs in stress granules remains unchanged. This could be due to compensation by the RNA helicase repertoire in cells. While DHX36 is thought to be the predominant RNA helicase that unwinds rG4, other RNA helicases such as BLM [27], DDX1 [57], DDX21 [58], and DHX9 [59] have also been demonstrated to resolve rG4s. Thus, suppression of DHX36 could be compensated through functional redundancy with other RNA helicases.

Moreover, when we co-treated DHX36 hypomorph cells with cPDS to further promote rG4 structure formation, we also did not observe any stress granule enrichment in our candidate mRNAs with putative rG4 motifs (Fig. 3C). Notably, these mRNAs exhibited a tendency to be excluded from stress granules under conditions that favor structure formation (Fig. 3C). Our data are consistent with recent observations that despite an increase in RNA secondary structures during cellular stress, more structured mRNAs are not enriched inside stress granules [60].

As such, our results support the model in which stress granule accumulation is driven by multiple promiscuous and synergistic RNA–RNA, RNA–protein, and protein–protein interactions that are largely independent of rG4 structure formation. Previous reports of rG4-binding proteins localized to stress granules might rely on linear G-rich sequences, alternative RNA conformations, or transient RNA structures. Instead of regarding rG4 as a default RNA structure, it could be more helpful to view rG4 folding as a highly regulated and conditional event [41]. Future work is required to understand the exact cellular conditions under which rG4 structures efficiently form and are functional.

While DHX36 did not affect the accumulation of the tested putative rG4-forming mRNAs into stress granules, we observed an effect of DHX36 on the kinetics of stress granule formation. First, by measuring stress granule assembly and disassembly kinetics, we observed that cells depleted of DHX36 formed more stress granules at a faster rate and dissolved stress granules more slowly than WT cells (Fig. 4), consistent with previous work [27]. Second, in G3BP1/2 dKO cells that do not form canonical stress granules [8], a reduction in DHX36 expression, via either siRNA-mediated knockdown or Cas9 targeting, rescued the formation of stress granule-like foci in a subset of cells (Fig. 5). These observations indicate that DHX36 contributes to limiting the formation of stress granules.

DHX36 might limit stress granules by two general mechanisms.

First, DHX36 could limit the formation of rG4s in *trans*, thereby reducing the number of intermolecular rG4s that stabilize stress granule assembly. This is because stress granules contain a high concentration of ribosome-free RNAs. These exposed RNA molecules have increased propensity to engage in intermolecular RNA–RNA interactions, which are thought to be important contributors in facilitating granule assembly by increasing RNA multivalency and enhancing scaffolding with other RNAs and/or RNA-binding proteins [1, 3, 4]. Two additional observations are consistent with *trans* rG4s supporting RNA condensation into stress granules. First, all four homopolymers can undergo RNA condensation *in vitro*, demonstrating that non-Watson–Crick interactions can drive RNA condensation [12]. Second, we observed that the addition of cPDS, whose only known biochemical activity is to stabilize rG4s, could partially rescue stress granule formation in G3BP1/2 dKO cell lines (Supplementary Fig. S5D).

Alternatively, DHX36 may also act on other types of intermolecular RNA–RNA or RNA–protein interactions that do not involve rG4 motifs to promote stress granule formation. This possibility is suggested by the observations that DHX36 has been shown to unwind DNA [26, 61] and RNA [62] duplexes—albeit with significantly lower efficiency compared to G4 structures. Moreover, *in vivo* SHAPE probing of WT and DHX36 KO HEK293T cells has demonstrated that DHX36 can remodel RNA secondary structures on a transcriptome-wide scale [56]. In the context of stress granules, where RNA crowding and high local concentrations favor the formation of diverse secondary structures, these alternative interactions may predominate due to their relative ease of formation. These findings suggest that DHX36 could play a broader role in modulating RNA structural dynamics within stress granules beyond its established activity on rG4s.

In either case, the role of DHX36 in limiting stress granule formation lends support for the “RNA chaperone network” model, where RNP granule assembly is thought to be regulated by a network of RNA-binding proteins, especially RNA helicases, that work to influence various RNA–RNA and RNA–protein interactions [3]. Previously, DEAD-box proteins eIF4A, DDX6, DDX3X, and the abundant monomeric RNA-binding protein YB1 have been shown to function in a type of RNA chaperone network to limit stress granule formation [27, 32, 34, 63, 64]. Our study suggests that DHX36 also acts to limit stress granule formation, although future experiments will be required to determine the specific mechanisms of DHX36 function. In addition, it will be important to investigate whether different human cell types have similar or distinct levels of DHX36 and other RNA helicases.

One limitation of our study is the absence of a direct measure of the fraction of G-tract sequences that are folded into rG4 structures in various mRNAs under different cellular conditions. While numerous techniques for assessing rG4 folding in cells have been developed, their sensitivity remains limited, particularly for detecting rG4 structures that are folded transiently or in a fraction of the transcripts. We plan to address this limitation in future studies.

In summary, our study offers an alternative perspective on the functional role of putative rG4s in RNA granules and provides additional support for the RNA chaperone network in regulating stress granule assembly. Considering the current perspectives on the role of G-tracts in RNA granules, additional rigorous studies will help evaluate the functional significance of such RNA motifs in their native cellular contexts.

## Supplementary Material

gkaf938_Supplemental_Files

## Data Availability

The data underlying this article will be shared on reasonable request to the corresponding authors.

## Notes

Present address: Regensburg Center for Biochemistry (RCB), University of Regensburg, 93053 Regensburg, Germany
